# Supplementary material for: Transcriptome Analysis and Identification of Genes Associated with Starch Metabolism in Castanea henryi Seed (Fagaceae)
Source: Int J Mol Sci. 2020 Feb 20;21(4):1431. doi: 10.3390/ijms21041431 (PMC7073145; doi:10.3390/ijms21041431)
Supplement: Supplementary file 1 [file ijms-21-01431-s001.pdf]

Supplementary Information

Supplementary Figures

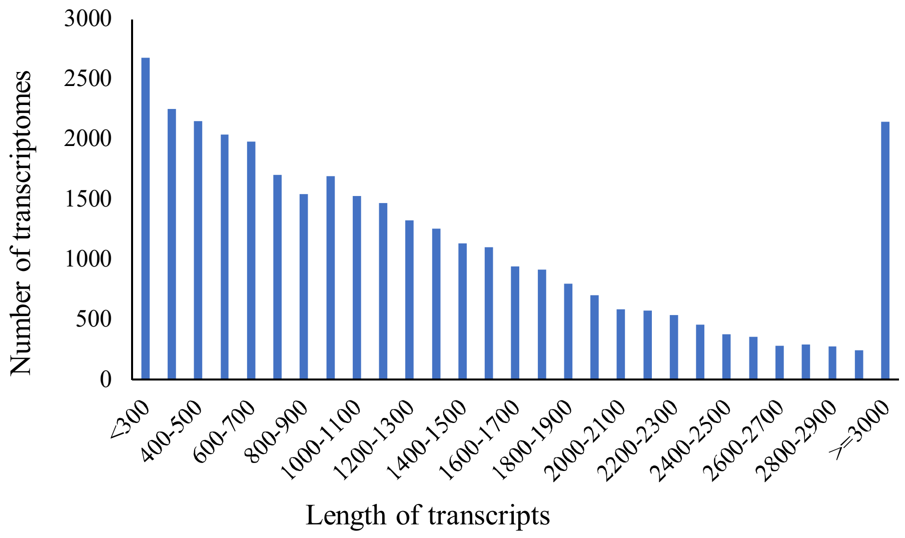

Supplementary Figure. S1. Distribution of transcripts lengths.

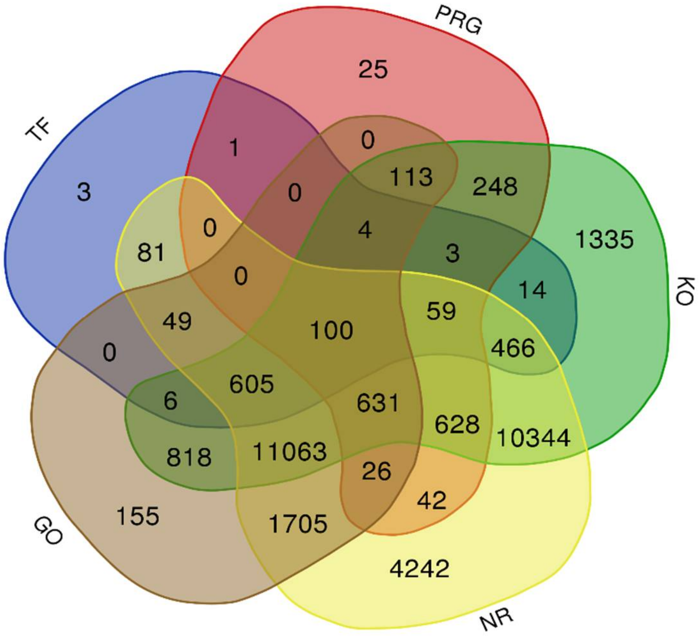

Supplementary Figure. S2. Venn diagram of the genes annotation results.

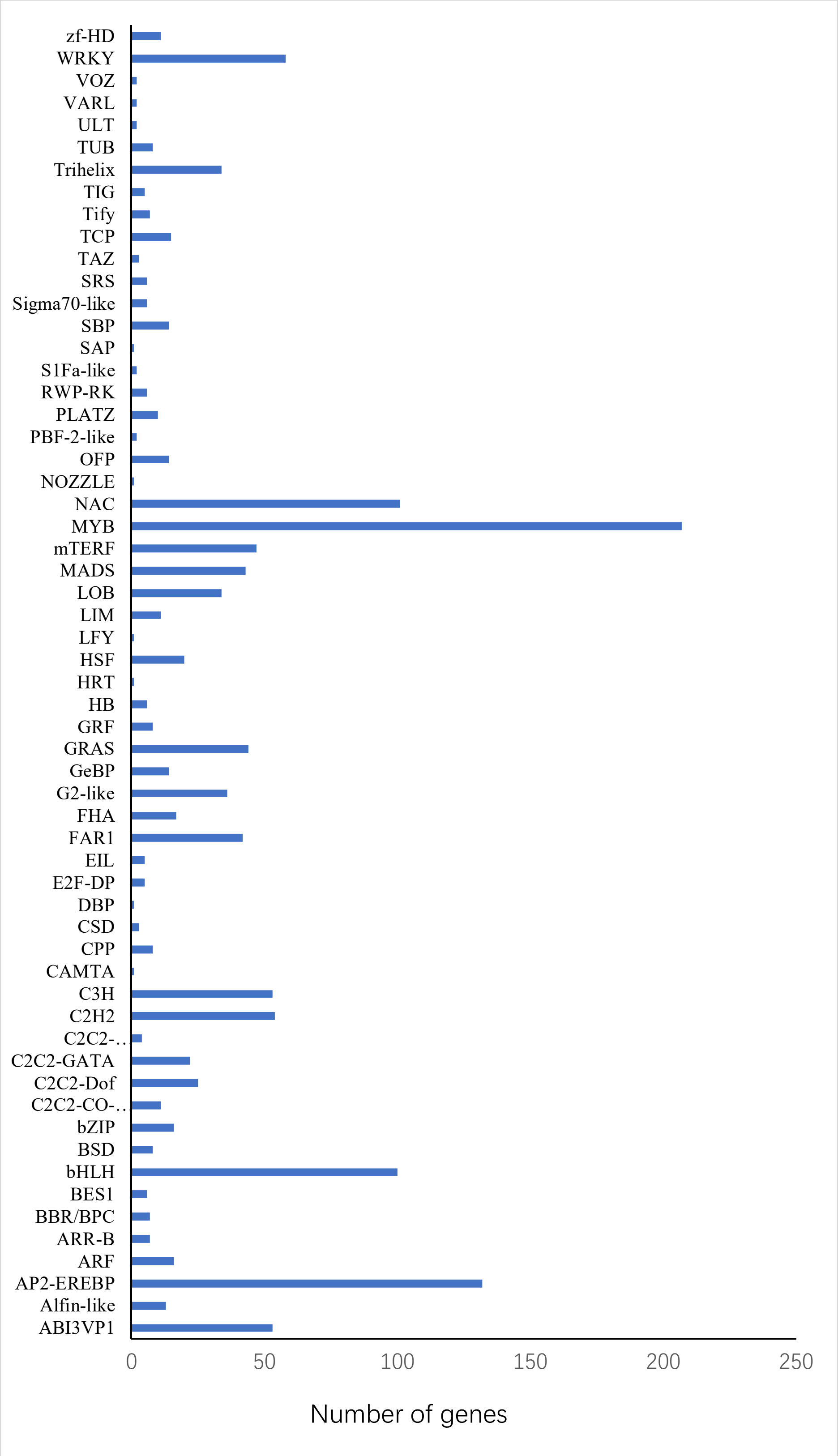

Supplementary Figure. S3. TF annotation of genes.

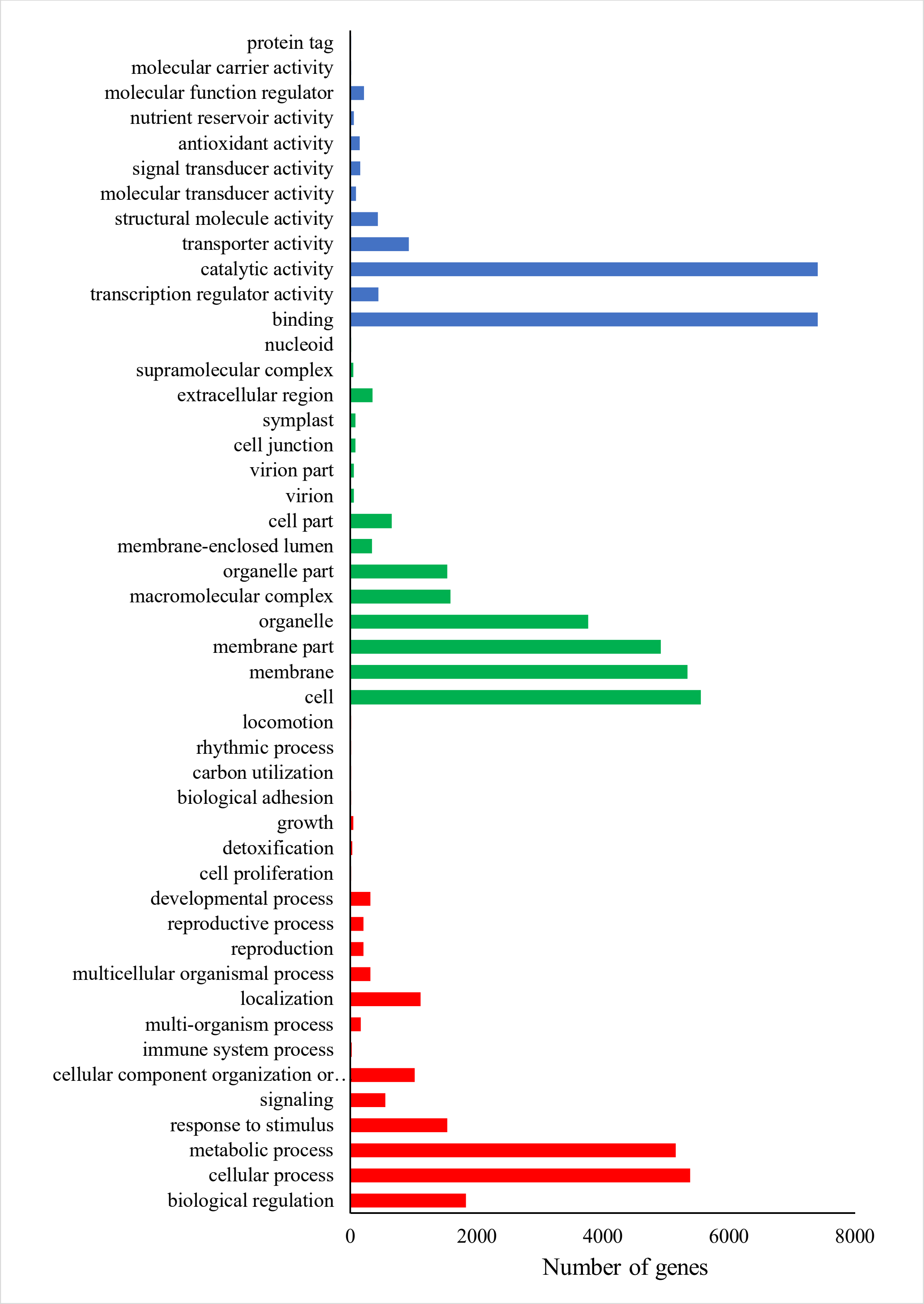

Supplementary Figure. S4. GO annotation of genes.

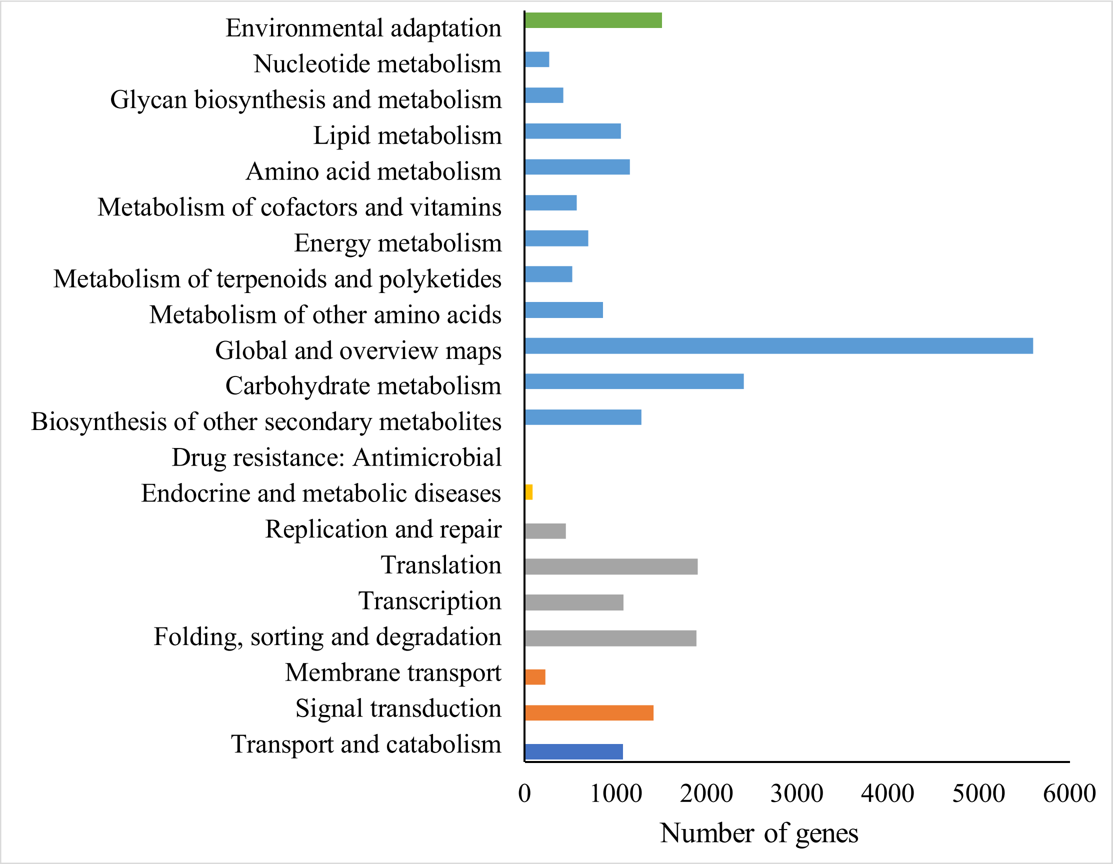

Supplementary Figure. S5. KEGG annotation of genes.

Supplementary Tables

Supplementary Table S1. RNA sequencing results and reference genome mapping statistics.

Sample	Raw Reads (M)	Clean Reads (M)	Reads Q20(%)	Total Mapping(%)	Uniquely Mapping(%)
T01-1	70.19	66.04	97.2	82.32	54.19
T01-2	67.68	63.76	97.34	82.4	54.41
T01-3	70.19	65.98	97.33	82.22	54.3
T02-1	67.68	62.47	97.03	81.41	52.68
T02-2	70.19	65.15	97.22	81.67	53.08
T02-3	70.19	65.12	97.02	81.85	52.97
T03-1	70.19	65.18	97.04	80.43	51.77
T03-2	67.69	62.77	97.06	80.3	51.45
T03-3	70.19	64.77	97.12	80.31	51.78
T04-1	70.19	64.65	97.16	80.52	53.17
T04-2	65.7	60.64	97.14	80.89	52.73
T04-3	67.68	62.5	97.16	80.73	52.76

Supplementary Table S2. The length distribution of assembled transcripts.

Sequence Size(nt)	Transcript Number	%
<300	2677	8.04%
300-500	4399	13.20%
500-1000	8955	26.88%
1000-2000	11168	33.52%
2000-3000	3973	11.93%

3000+	2142	6.43%
Total number	33314	

Supplementary Table. S3. Annotation of genes were searched against TF, PRG, GO, KEGG and Nr databases.

Database	Number of Genes	Percentage of All Genes (%)
TF	1391	3.79
PRG	1880	5.12
GO	15275	41.58
KEGG	26437	71.97
Nr	30041	81.78
Annotated	32766	89.20
Total genes	36734	

Supplementary Table S4. Highly and differentially expressed genes involved in starch and sucrose metabolism in *C. henryi* seeds.

Description	Gene ID	T01_FPKM	T02_FPKM	T03_FPKM	T04_FPKM
AGPase	Che003775	27.84	11.87	7.96	8.80
	Che007374	600.76	490.82	433.73	364.25
	Che013145	305.95	180.45	120.53	199.51
NPPS	Che006880	26.96	15.28	19.65	17.71
	Che018517	21.03	108.91	99.69	149.35
	Che032493	72.77	38.58	38.47	41.62
SS	Che013592	94.21	32.62	37.55	40.39
	Che022142	156.55	66.45	61.14	61.91
	Che022683	16.90	8.96	9.72	9.68
	Che035698	0.05	25.51	70.05	6.02
	Che036389	54.81	16.93	20.21	20.58
GBSS	Che000347	17.69	8.79	6.95	7.83
	Che029279	3019.21	3654.08	5289.10	4315.59
SBE	Che001890	66.53	91.34	83.95	122.89
	Che014648	26.80	10.38	9.56	7.70
	Che040552	587.73	661.23	969.03	903.45
AMY	Che011855	36.98	13.29	14.00	18.84
	Che011856	52.93	48.13	80.21	143.65
	Che011857	159.28	328.26	290.06	305.64
	Che034190	83.43	89.43	118.66	190.39
BMY	Che000244	21.30	6.16	6.21	4.20
	Che003213	51.51	7.66	7.02	24.88
	Che009477	344.00	40.57	55.47	53.60
	Che013300	8.49	30.00	76.86	66.23
	Che017448	41.50	207.88	187.52	249.53
	Che021344	305.22	598.71	1055.16	955.90
SP	Che005965	63.82	34.41	45.80	45.73
	Che016246	333.05	477.33	697.74	188.40
	Che025487	45.85	152.81	473.49	361.91
ISA	Che017410	5.16	12.36	15.79	18.37
	Che019232	116.20	50.72	87.77	56.64
MAL	Che005471	17.46	94.82	75.40	90.31
	Che005472	11.57	44.89	49.32	51.15
	Che010778	63.44	48.74	40.78	29.08

	Che015252	12.24	68.84	73.75	31.98
	Che015253	10.93	67.92	67.68	25.83
UGPase	Che002934	201.94	233.93	232.26	173.75
	Che009576	6.82	1.39	0.67	1.02
	Che032936	13.83	3.14	3.02	1.47
SuSy	Che028628	0.02	67.47	3.75	53.09
	Che028629	80.98	672.63	279.23	213.89
SPS	Che002155	25.65	14.82	15.47	7.97
SPP	Che022264	125.89	34.14	45.56	42.48
INV	Che001882	0.66	8.84	5.64	6.28
	Che009013	1.85	44.53	36.12	32.23
	Che019953	12.43	18.89	11.37	6.20
	Che025004	14.49	4.48	2.84	3.43
	Che036493	37.58	38.72	47.75	46.25
	Che036502	7.57	0.63	0.20	0.65

Supplementary Table S5. Primers used in this study.

Gene ID	Description	Sequence (5' - 3')	Primer Length/bp	Product Length/bp
Che006880	NPPS	AGTCAGAAGGAGGTTGTGGAA	21	232
		CATTACAGGTAGGCAGTGAAGT	22	
Che032493	NPPS	CAACTGAACCACGCAACTGAG	21	224
		TCTTCCAACTTCTGCCATACCA	22	
Che003775	AGPase	AGCAGATGCAGTGAGGCAATTC	22	191
		AATCTGATGCACGGCTGTTACC	22	
Che013145	AGPase	GCCAAAGAGACCCTGACCATAC	22	261
		GAACCACTTCATTCCTGCTTCC	22	
Che013592	SS	CCTCTGATGTTGACTCCACCAA	22	168
		TTGCTTCACCATCCACCTCTG	21	
Che022683	SS	TTCGCCTCCAAACCCAACCT	20	263
		ACTCCTCCACTGCCACAAGAC	21	
Che036389	SS	TCTCTACTCTCGCCTCAGAACC	22	219
		ATGGAGTCAACACAGACCTTCG	22	
Che034253	RP-L34e	GCTACGCCACCAAATCCAATC	21	115
		CTCTTGCCAGTTACAGGACACT	22	
